# Depression, Coping Strategies, Glycemic Control and Patient Compliance in Type 2 Diabetic Patients in an endocrine Outpatient Clinic

**DOI:** 10.12669/pjms.311.6011

**Published:** 2015

**Authors:** Hulya Parildar, Ozlem Cigerli, Nilgun Guvener Demirag

**Affiliations:** 1 Dr. Hulya Parıldar, Associate Professor, Department of Family Medicine, Başkent University Faculty of Medicine, Istanbul, Turkey.; 2 Dr. Ozlem Cigerli, Department of Family Medicine, Başkent University Faculty of Medicine, Istanbul, Turkey.; 3 Dr. Nilgun G Demirag, Professor, Department of Endocrinology and Metabolism, Başkent University Faculty of Medicine, Istanbul, Turkey.

**Keywords:** Diabetes Mellitus Type 2, Depression, Coping Skills, Diabetes Care

## Abstract

**Objective:**

Diabetes is a multifactorial disorder posing a great challenge to public health. In this study, we aimed to evaluate the relationship between depression, coping strategies, glycemic control and patient compliance in type 2 diabetic patients.

**Methods:**

Total 110 outpatients (mean (SD) age: 57.9 years (10.5), 56.4% were females) with type 2 diabetes mellitus were included in this descriptive and cross-sectional study. They were followed-up in the endocrinology outpatient clinic at Baskent University Istanbul Hospital Turkey. A questionnaire including items on sociodemographics, patient compliance, Beck Depression Inventory (BDI) and Ways of Coping Questionnaire (WCQ) were used. Glycemic control was measured by HbA1c levels.

**Results:**

Mean depression score was 12.6(9.2) with moderate to severe depression in 30.9% of study participants. Overall scores for BDI, fatalism and helplessness approaches were significantly higher among females compared with male patients. Depression scores were correlated positively to duration of disease (r=0.190, p=0.047), fatalistic (r=0.247, p=0.009), helplessness (r=0.543, p=0.000) and avoidance (r=0.261, p=0.006) approaches, and negatively to educational status (r=-0.311, p=0.001) and problem solving-optimistic approach (r=-0.381, p=0.000).

**Conclusions:**

Likelihood of depression was frequent, consistent with literature and was associated with gender, educational status, coping strategies, duration of diabetes and patient compliance with treatment in our study**. **Screening for depression and patient education may improve the quality of life in diabetic patients.

## INTRODUCTION

Data from clinical trials support the association between depression and hyperglycemia with the likelihood of a reciprocal interaction, as well as a gain in medical outcomes through long-term depression management in diabetic patients.^1^^,^^2^

Patients with diabetes are continuously challenged with the stressing demands of the disease and use different ways of approaches to cope with them.^3^ A crucial element of the treatment is self-care that depends on the patient’s own responsibility and this has been associated with personal psychological and social situation.^4^ Additionally, studies also suggest that in chronic diseases such as diabetes, the resultant poor health is a continuous source of stress besides everyday stressors, and should be appropriately managed.^3^ In this respect, diabetic patients seem to be affected by diabetes-related distress associated with daily treatment regimen and long-term complications of the disease which have independent negative impact on glycemic control and self-management.^2^^,^^3^ Accordingly, coping, an individual’s ability to constantly change their cognitive and behavioural efforts in managing stressful situations, according to Lazarus and Folkman’s (1984) Transactional Model of Stress, is a behavior considered in relation to management of diabetes self-care.^3^^,^^5^ Functioning as a mediator between stressful events and outcomes such as depression, several different coping strategies are known to operate together with the purpose of managing a stressful situation, including problem-focused coping (PFC) with problem-solving action, logical analysis, information gathering and seeking social support and emotion-focused coping (EFC) of self-blaming, wishful thinking and avoidance.^6^^-^^8^

Several guidelines for diabetes care recommend screening for depression, only 25% of depressed diabetic patients have been estimated to be diagnosed in clinical practice.^7^^-^^9^ Therefore, the present study was designed to evaluate the relationship between depression, coping strategies, metabolic control and compliance with diabetes care in diabetic outpatients in a tertiary hospital.

## METHODS

This descriptive, cross-sectional study included 110 type 2 diabetic patients who were followed-up in the endocrinology outpatient clinic at Baskent University Istanbul Hospital. Type 2 diabetic patients having mental and cognitive abilities required for answering the questionnaires were included. Patients with acute/chronic renal, hepatic and/or cardiac failure and malignancies, thyroid function abnormalities and DSM-IV based diagnosis of any psychiatric disorder and psychiatric treatment for a psychiatric disease, severe physical or mental retardation were excluded from the study. Since an important part of the impact of affective state in diabetic patients is related to the presence and stage of diabetic complications (retinopathy, nephropathy, cardiovascular disease, neuropathy, stroke etc), diabetics who had micro and/or macrovascular complications or any other chronic organ failure were also excluded. Informed consent was obtained from each subject following a detailed explanation of the objectives and protocol of the study which was conducted in accordance with the ethical principles stated in the “Helsinki Declaration”. This study was approved by Baskent University Institutional Review Board (No: KA12/230).

A questionnaire including items on sociodemographic features and patient compliance with diet & exercise and pharmacological treatment of diabetes as well as items related to Beck Depression Inventory for identification of depressive status and Ways of Coping Questionnaire (WCQ) to determine coping strategies among diabetic patients was administered via face to face interview method. 

The Beck Depression Inventory (BDI), a 21 item screening questionnaire comprising 13 cognitive and 8 somatic questions was used to assess the affective, motivational, cognitive and somatic symptoms of depression. Each item of the inventory scores ranging from 0-3 points indicates the severity of the depressive symptoms (total scores > 17 showing moderate to severe depressive symptoms). Studies have demonstrated that BDI is reliable and valid for Turkish population samples.^10^

WCQ, developed and later revised by Folkman and Lazarus, addresses a broad range of cognitive and behavioral strategies that individuals use when they encounter an internal and/or external stressful situation.^4^^,^^5^^,^^11^ In a study, 42-item version of the Turkish adaptation of the questionnaire by Karanci et al., which includes addition of eight new items about fatalism and superstition that were thought to be relevant to the Turkish culture, was used.^12^ For assessing coping process in our study WCQ was used and 4 different approaches (Problem Solving-optimistic, Fatalism, Helplessness and Avoidance) were evaluated in relation to depression and anxiety.

Metabolic control was measured by glycosylated hemoglobin A1c (HbA1c) levels (%) known as a reliable immunoturbidimetric method for estimating glycemic control over the last 90-120 days.

Statistical analysis was made using SPSS version 11.0 (SPSS Inc. Chicago, IL, USA). Independent sample t-test and Chi-square test were used for the comparison of numeric and categorical variables, respectively. Correlations were evaluated via Spearman correlations analysis. Data were expressed as “mean (standard deviation; SD)” and percentage (%) where appropriate. For the level of significance, p<0.05 was considered statistically significant.

## RESULTS

***Demographic and baseline clinical features: ***Among 110 patients with DM2 (mean age was 57.9 (10.5) years, 56.4% female), 59.1% had diabetes for less than 5 years while new-onset diabetes was identified in 33.6% of patients. Family history of diabetes mellitus was evident in 11.8% of study population.

Moderate to very good compliance with diet and pharmacological treatment was noted in 87.3 and 91.0% of patients, respectively. Lack of regular physical exercise was determined in 21.8% while lack of patient education on diabetes was recorded in 32.7% of patients (Table-I).

Almost half of the patients (48.2%) reported that they visit their doctor in every 1-2 months and 36.4% of them reported that they measure their blood glucose levels 4-8 times per week by blood glucose meters. Diabetes treatment was based on oral antidiabetics (OADs) in 50.9%, insulin in 20.9% and OADs + insulin in 20.9% (Table-I).

Mean scores for BDI was 12.6(9.2) with moderate to severe depression in 30.9% of the overall population. The mean scores were 2.3(0.4) for problem solving-optimistic and 2.2(0.5) for fatalistic approaches followed by avoidance (2.0(0.3)) and helplessness (1.9(0.4)) scores. Mean HbA1c (%) value was 7.48(SD:1.7, range: 5.3 to 14.8).(Table-II).

Overall scores for BDI (p<0.01), fatalism (p<0.01) and helplessness (p<0.01) approaches were significantly higher among females compared with males (Table-II).

Compliance to diet and exercise was better in patients without depression compared to those with depression (p=0.033 and p=0.024, respectively). The percentage of patients with or without depression was similar among patients with poor compliance to pharmacological treatment (50.0% for each, p=0.470) (Table-III).

Depression scores were positively correlated to the duration of disease (r=0.190, p=0.047) while negatively correlated to the educational status (r=-0.311, p<0.01) (Table IV).There was no significant difference between marital status (77.2% (n=85) were married) and depression (p=0.445) and ways of coping (problem solving p=0.548, fatalistic p=0.308, helplessness p=0.632, avoidance p=0.401).

Problem solving approach was positively correlated (r=0.307, p<0.01) while fatalistic approach (r=-0.454, p<0.01), helplessness (r=-0.287, p<0.01) and avoidance (r=-0.210, p=0.029) were negatively correlated to educational status. Helplessness approach (r=0.191, p=0.045) was positively correlated to duration of the disease (Table-IV).

There was no significant correlation between HbA1c levels and depression or coping strategies. Depression scores were positively correlated to fatalistic approach (r=0.247, p<0.01), helplessness (r=0.543, p<0.01) and avoidance (r=0.261, p<0.01) while they were negatively correlated to problem solving approach (r=-0.381, p<0.01) 

## DISCUSSION

Our findings revealed the presence of depressive symptoms in 55.5% of our study population compared to established (64.4%) or newly diagnosed (33.6%) type 2 diabetic patients having good compliance with diet (90.9%) and pharmacological treatment (96.4%). The likelihood of depression among diabetic patients was associated with gender, educational status, duration of diabetes, coping strategies, compliance with diet and exercise, but not with the current metabolic control and compliance with pharmacological treatment.

Previous studies revealed an increase in depressive symptoms in 30–40% of diabetic patients, minimal to mild depression in 28 to 44% and confirmed depressive disorder according to clinical criteria in 10 to 15%.^13^^,^^14^ Our findings showed that 55.5% of our patients were suffering from depressive symptoms with moderate to severe depression in 30.9% of them and this is slightly higher than the published data.^8^ This finding can be explained by some characteristics of our study population including a higher percentage of female patients and lower educational status. Also, admission of problematic cases with a higher incidence of depression in a tertiary hospital like ours is also likely.

**Table-I:** Patient demographics and basic clinical features (N=110).

Age (years) Mean(SD)	57.9 (10.5)	Treatment type	n (%)
Gender	n (%)	Diet per se	8(7.3)
Female	62 (56.4)	OADs	56(50.9)
Male	48 (43.6)	Insulin	23(20.9)
Marital status	n (%)	Insulin + OAD	23(20.9)
Married	85(77.3)	Compliance with diet	n (%)
Single	3(2.7)	Good-Very good	67(60.9)
Widow or widower	16(14.5)	Moderate	33(30.0)
Divorced	6(5.5)	Poor	10(9.1)
Educational status	n (%)	Compliance with pharmacological treatment	n (%)
Literate	6(5.5)	Good-Very good	94(85.5)
Primary school	60(54.5)	Poor	16(14.5)
High school	13(11.8)	Physician visits	n (%)
University	31(28.2)	once per 1-2 months	53(48.2)
Diagnosis of diabetes	n (%)	once per 3-6 months	41(37.3)
Prior diagnosis	71(64.5)	yearly	9(8.2)
New diagnosis	39(35.5)	less than once per year	7(6.4)
Duration of diabetes	n (%)	Blood glucose measurement	n (%)
≤1 year	37(33.6)	everyday	19(17.3)
1-5 years	28(25.5)	4-8 times/week	40(36.4)
6-10 years	15(13.6)	4-8 times/15 days	20(18.2)
≥10 years	30(27.3)	2-4 times/month	21(19.1)
	n (%)	less than 2-4 times/month	10(9.1)
Regular physical exercise	47(42.7)		
Family history of diabetes	13(11.8)		
Patient education on DM	73(66.4)		

DM: diabetes mellitus, OAD: oral antidiabetic drug, SD: standard deviation.

**Table-II:** Average scores for depression, coping strategies and HbA1c

	Total	Males	Females	p value
Coping strategy	Mean(SD)			
Problem solving-optimistic approach	2.3(0.4)	2.3(0.4)	2.2(0.3)	0.426
Fatalistic approach	2.2(0.5)	2.0(0.5)	2.4(0.4)	0.000
Helplessness approach	1.9(0.4)	1.7(0.4)	2.0(0.4)	0.002
Avoidance approach	2.0(0.3)	1.9(0.3)	2.1(0.3)	0.054
	Mean(SD);			
HbA1c	7.4(1.7); (range 5.3- 14.8)	7.5(1.9)	7.3(1.5)	0.553
Beck depression inventory scores	Mean(SD)			
Overall	12.6(9.2)	9.3(7.5)	15.1(9.6)	0.001
Distribution	Total n(%)	Males n(%)	Females n(%)	
*No depression (scores <10)*	49(44.5)	28(58.3)	21(33.9)	
*Mild depression (scores 10-16) *	27(24.5)	9(18.8)	18(29)	
*Moderate depression (scores 17-29) *	28(25.5)	11 (22.9)	17(27.4)	
*Severe depression (scores ≥30)*	6(5.5)	0(0)	6(9.7)

**Table-III:** The association between compliance with lifestyle modification and pharmacological treatment and depression scores

	Depression			p value
Compliance with diet	Absent	Present	Total	
Good	35(52.2)	32(47.8)	67(60.9)	X^2^=4.107 p=0.033
Poor	14(32.5)	29(67.5)	43(39.1)	
Total	49(44.6)	61(55.4)	110(100.0)	
*Compliance with pharmacological treatment*				
Good	44(44)	56(56)	100 (90.9)	X^2^=0.132 p=0.484
Poor	5(50.0)	5(50.0)	10 (9.1)	
Total	49(44.5)	61(55.5)	110(100.0)	
*Compliance with exercise*				
Good	27(55.1)	21(44.9)	49 (44.5)	X^2^=4.723 p=0.024
Poor	22(36.0)	40 (64.0)	61 (55.5)	
Total	49(44.5)	61(55.5)	110(100.0)

**Table-IV:** Correlations between psychometric scores, patient compliance, HbA1c levels, educational status and duration of diabetes mellitus

		BDS	PSOA	FA	HA	AA	HbA1c (%)	Education	DM duration
BDS	r p	1.000 -	-0.381** 0.000	0.247* 0.009	0.543** 0.000	0.261** 0.006	-0.039 0.684	-0.311** 0.001	0.190 0.047*
PSOA	r p		1.000 -	0.187 0.051	-0.169 0.077	0.101 0.293	0.058 0.547	0.307** 0.001	0.006 0.949
FA	r p			1.000 -	0.297** 0.002	0.325** 0.001	-0.005 0.962	-0.454** 0.000	0.060 0.531
HA	r p				1.000 -	0.347** 0.000	-0.096 0.320	-0.287** 0.002	0.191* 0.045
AA	r p					1.000 -	-0.33 0.735	-0.210* 0.029	0.088 0.359
HbA1c (%)	r p						1.000 -	-0.081 0.579	0.130 0.375
Education	r p							1.000 -	-0.051 0.603
DM duration	r p								1.000 -

AA: Avoidance approach, BDS: Beck Depression Score, FA: fatalistic approach, HA: helplessness approach, DM: diabetes mellitus, PSOA: problem solving-optimistic approach

* significant at the 0.05 level (2-tailed);

** significant at the 0.01 level (2-tailed).

Several factors such as gender, being unmarried, low education level, higher number of comorbidities and complications, and the need for insulin treatment have been associated with higher incidence of depression during the course of diabetes mellitus.^15^ According to our findings, depression was more likely in females, in patients using fatalistic, helplessness and avoidance approaches, in patients with poor compliance with dietary recommendations, lower educational status and longer duration of diabetes mellitus as consistent with the literature. Gender differences in diabetes complications have been suggested to occur as a result of biological differences in coping efforts that affect diabetes self-care.^12^ Indeed, the prevalence of depression among diabetics has been studied in different surveys and an association with female gender has been previously reported.^12^^,^^16^^-^20 Accordingly, in line with the positive correlations of fatalistic and helplessness approaches with depression scores, there was a significant gender influence on depression scores and coping strategies in our study population with significantly higher likelihood of depression as well as use of fatalistic and helplessness approaches among females. Supporting the fact that men use more problem-solving strategies, while women have more social and emotional aspects integrated into their coping strategies, our finding may emphasize different psychosocial needs necessitating somewhat different diabetes care for men and women.^18^^,^^19^

Lesser likelihood of depressive symptoms in patients using problem solving-optimistic approach in our study is compatible with the identification of problem-solving skill, along with problem-solving orientation, disease specific knowledge, and transfer of past experience as key components of effective self-management. Likewise, an increased rate of depression was reported in adult diabetics who tend to use emotion focused coping (EFC) than diabetics who tend to use more problem focused coping (PFC) strategies compatible with the fact that PFC was related negatively and EFC was related positively to depression.^7^^,^^20^

Positive correlation between depression scores and duration of diabetes in this study is in line with the statement that data on psychosocial problems in diabetes often were derived from studies on patients with long disease duration.^2^^,^^4^ However, as diabetes was newly diagnosed in one third of our study population, it seems even more important to identify patients with psychosocial problems early in the course of the disease. This would enable the prompt implementation of helpful psychosocial interventions to manage the negative physical and social consequences.^2^^,^^4^

Depression-related risk of developing DM2 was previously reported to be higher in low educational status irrespective of age, gender, race, health behaviors, BMI, and family history of diabetes.^17^^,^^18^^,^^20^ Besides, once diagnosed, depression was shown to be recurrent in diabetic patients with higher likelihood of suffering from persistent depressive disturbances among less educated patients.^17^^,^^20^ In this regard, since lower educational status was associated with higher incidence of depressive symptoms and more common use of fatalistic and helplessness approaches, identification of depressive symptoms in more than half of the study population may also be associated with the fact that 54.5% of our patients were primary school graduates. Educational status was the single most important factor associated both with depression scores and all types of coping strategies in our study population, emphasizing the crucial role of patient education on self-management of diabetes mellitus, as well as the role of using more active coping strategies in management of DM2 and comorbidities.

Indeed, from the patient perspective, the challenges associated with having diabetes have been reported to be dynamic rather than static which emphasize the role of coping with emerging issues rather than simple “education” about standard responses or regimen behaviors.^21^

Unlike previous studies, we found no significant association between HbA1c levels and psychometric variables including depression and coping strategies.^22^ In a study there was no significant correlation between the severity of depression and HbA1c levels.^23^ In a review, it was suggested that pharmacological treatments for depression improve mood, but have little effects on glycemic control in diabetes.^24^ Since our study was designed as cross-sectional, we were not able to determine whether depression preceeded diabetes or not. Indeed, patients’ subjective symptom perception of diabetes was related to depressed mood rather than to objective measures of glycemic control.^17^^,^^20^ Likewise, analysis for depression predictors in diabetic patients has shown that psychological factors are more strongly associated with development of depressive symptoms rather than the disease-associated variables.^1^

Major limitations of our study are the small sample size and the use of self-reported information on the compliance with diet and pharmacological treatment as the outcome measure. And its cross sectional design that we used does not enable us determine the directionality or causality. In addition, diagnosis of depression was based on subjective scoring of depressive symptom severity via Beck Depression Inventory rather than structured interviews, therefore possibility of misdiagnosed depression influencing our findings remains.

Our findings emphasize the importance of routine screening for symptoms of depression among diabetic patients, patient education on self-management of diabetes mellitus and counseling for encouraging the use of problem-oriented coping strategies for the successful management of patients with diabetes and comorbid disorders early in the course of this disease.

## Authors Contribution:

**HP:** General design, writing the manuscript, data acquisition, interpretation, final approval of the version.

**OC:** The statistical analysis and final approval of the manuscript.

**NGD:** General design, interpretation, final approval of the manuscript.

## References

[1] Pouwer F, Kupper N, Adriaanse MC (2010). Does emotional stress cause type 2 diabetes mellitus? A review from the European Depression in Diabetes (EDID) Research Consortium. Discov Med.

[2] Lustman PJ, Clouse RE (2005). Depression in diabetic patients: the relationship between mood and glycemic control. J Diabetes Complications.

[3] Karlsen B, Idsoe T, Dirdal I, Rokne Hanestad B, Bru E (2004). Effects of a group-based counseling programme on diabetes-related stress, coping, psychological well-being and metabolic control in adults with type 1 or type 2 diabetes. Patient Educ Couns.

[4] Rane K, Wajngot A, Wändell PE, Gåfvels C (2011). Psychosocial problems in patients with newly diagnosed diabetes: number and characteristics. Diabetes Res Clin Pract.

[5] Lazarus RS, Folkman S (1984). Stress, appraisal and coping.

[6] Clarke D, Goosen T (2009). The mediating effects of coping strategies in the relationship between automatic negative thoughts and depression in a clinical sample of diabetes patients. Pers Individ Diff.

[7] Vitaliano PP, Russo J, Carr JE, Becker M&J (1985). The Ways of Coping Checklist: Revision and psychometric properties. Multivariate Behav Res.

[8] Karlsen B, Oftedal B, Bru E (2012). The relationship between clinical indicators, coping styles, perceived support and diabetes-related distress among adults with type 2 diabetes. J Adv Nurs.

[9] Petrak F, Herpertz S, Albus C, Hirsch A, Kulzer B, Kruse J (2005). Psychosocial factors and diabetes mellitus: evidence based treatment guidelines. Curr Diabetes Rev.

[10] Aydemir O, Koroglu E (2000). Symptom Checklist (SCL-90-R) in Clinical Scales in Psychiatry [Psikiyatride Kullanilan Klinik Ölçekler], Ankara, Turkey.

[11] Folkman S, Lazarus RS (1988). Coping as a mediator of emotion. J Pers Soc Psychol.

[12] Karanci NA, Alkan N, Aksit B, Sucuoglu H, Balta E (1999). Gender differences in psychological distress, coping, social support and related variables following the 1995 Dinar (Turkey) earthquake. N Am J Psychol.

[13] Hermanns N, Kulzer B, Krichbaum M, Kubiak T, Haak T (2006). How to screen for depression and emotional problems in patients with diabetes: comparison of screening characteristics of depression questionnaires, measurement of diabetes-specific emotional problems and standard clinical assessment. Diabetologia.

[14] Anderson RJ, Freedland KE, Clouse RE, Lustman PJ (2001). The prevalence of comorbid depression in adults with diabetes: a meta analysis. Diabetes Care.

[15] Katon W, von Korff M, Ciechanowski P, Russo J, Lin E, Simon G et al (2004). Behavioral and clinical factors associated with depression among individuals with diabetes. Diabetes Care.

[16] Lynch CP, Egede LE (2011). Optimizing diabetes self-care in low literacy and minority population problem-solving, empowerment, peer support and technology-based approaches. J Gen Intern Med.

[17] Lustman PJ, Griffith LS, Clouse RE (1988). Depression in adults with diabetes: Results of a 5-yr follow-up study. Diabetes Care.

[18] Chiu CJ, Wray LA (2011). Gender differences in functional limitations in adults living with type 2 diabetes: biobehavioral and psychosocial mediators. Ann Behav Med.

[19] Glasgow RE, Fisher L, Skaff M, Mullan J, Toobert DJ (2007). Problem solving and diabetes self-management: investigation in a large, multiracial sample. Diabetes Care.

[20] Mezuk B, Eaton WW, Golden SH Golden SH, Ding Y (2008). The Influence of Educational Attainment on Depression and Risk of Type 2 Diabetes. Am J Public Health.

[21] Peyrot M, Rubin RR (1999). Persistence of depressive symptoms in diabetic adults. Diabetes Care.

[22] DeGroot M, Anderson R, Freedland KE, Clouse RE, Lustman PJ (2001). Association of depression and diabetes complications: a meta-analysis. Psychosom Med.

[23] Sevincok L, Guney E, Uslu A, Baklaci F (2001). Depression in a sample of Turkish type 2 diabetes patients. Eur Psychiatry.

[24] Egede LE, Ellis C (2010). Diabetes and depression: Global perspectives. Diabetes Res Clin Pract.

